# Accelerating Bayesian Hierarchical Clustering of Time Series Data with a Randomised Algorithm

**DOI:** 10.1371/journal.pone.0059795

**Published:** 2013-04-02

**Authors:** Robert Darkins, Emma J. Cooke, Zoubin Ghahramani, Paul D. W. Kirk, David L. Wild, Richard S. Savage

**Affiliations:** 1 Systems Biology Centre, University of Warwick, Coventry, United Kingdom; 2 Department of Chemistry, University of Warwick, Coventry, United Kingdom; 3 Department of Engineering, University of Cambridge, Cambridge, United Kingdom; University of Manchester, United Kingdom

## Abstract

We live in an era of abundant data. This has necessitated the development of new and innovative statistical algorithms to get the most from experimental data. For example, faster algorithms make practical the analysis of larger genomic data sets, allowing us to extend the utility of cutting-edge statistical methods. We present a randomised algorithm that accelerates the clustering of time series data using the Bayesian Hierarchical Clustering (BHC) statistical method. BHC is a general method for clustering any discretely sampled time series data. In this paper we focus on a particular application to microarray gene expression data. We define and analyse the randomised algorithm, before presenting results on both synthetic and real biological data sets. We show that the randomised algorithm leads to substantial gains in speed with minimal loss in clustering quality. The randomised time series BHC algorithm is available as part of the R package *BHC*, which is available for download from Bioconductor (version 2.10 and above) via http://bioconductor.org/packages/2.10/bioc/html/BHC.html. We have also made available a set of R scripts which can be used to reproduce the analyses carried out in this paper. These are available from the following URL. https://sites.google.com/site/randomisedbhc/.

## Introduction

Many scientific disciplines are becoming data intensive. These subjects require the development of new and innovative statistical algorithms to fully utilise these data. Time series clustering methods in particular have become popular in many disciplines such as clustering stocks with different price dynamics in finance [1], clustering regions with different growth patterns [2] or signal clustering [3].

Molecular biology is one such subject. New and increasingly affordable measurement technologies such as microarrays have led to an explosion of high-quality data for transcriptomics, proteomics and metabolomics. These data are generally high-dimensional and are often time-courses rather than single time point measurements.

It is well-established that clustering genes on the basis of expression time series profiles can identify genes that are likely to be co-regulated by the same transcription factors [4]. There have been a number of approaches developed to clustering time series, for example using finite or infinite hidden Markov models [5], [6]. Another popular approach is the use of splines as basis functions [7]–[10]. [11] also use Fourier series as basis functions. A number of additional methods for time series data analysis have been reviewed by [12].

These statistical methods often provide superior results to standard clustering algorithms, at the cost of a much greater computational load. This limits the size of data set to which a given method can be applied in a given fixed time frame. Fast implementations of the best statistical methods are therefore highly valuable.

The Bayesian Hierarchical Clustering (BHC) algorithm has proven a highly successful tool for the clustering of microarray data [13]–[15]. The time series BHC method uses Gaussian processes to model time series in a flexible way, making the method highly adaptive and able to handle a wide range of structure in the data.

The principal downside of the BHC algorithm is its run-time, in particular its scaling with the number of items clustered. This can be addressed via *randomised algorithms* [16], a class of techniques that can be highly powerful in this regard. Randomised algorithms employ a degree of randomness as part of their logic, aiming to achieve good average case performance with high probability. Because the requirement for guaranteeing a certain (e.g. optimal) result is relaxed, it is often possible to obtain significantly improved performance as a result.

In this paper, we apply the approach of [17] to create a randomised BHC algorithm for clustering microarray time series. This allows much larger time series data sets to be analysed in a given amount of time, substantially extending the utility of the time series BHC method.

## Results

### Synthetic Data Results

To demonstrate the effectiveness of the randomised BHC algorithm, we test its performance on a realistic synthetic data set. We use synthetic data constructed from several realisations of the *S. cerevisiae* synthetic data generated in [15]. Using the fact that Gaussian processes are generative models, we draw random realisations from the BHC model obtained on a 169-gene subset of the cell cycle gene expression data of [18], to give a total of 1000 genes, spread across 13 distinct clusters.

Given that for these synthetic data we know the ground truth clustering partition, we use the adjusted Rand index as our performance metric [19].

**Figure 1:**
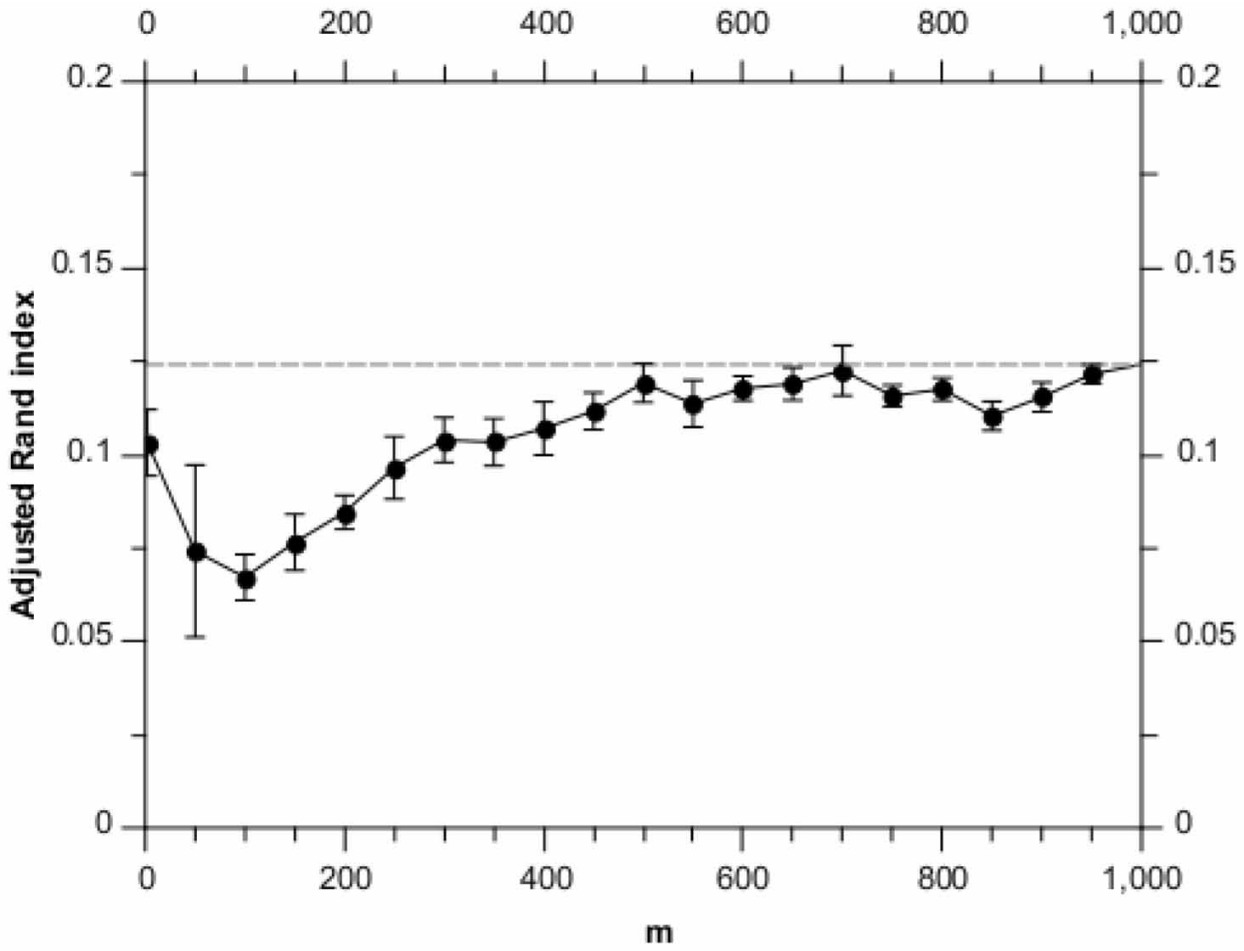
Adjusted Rand index scores for different values of 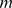, analysing the synthetic data set. Each point is the average of 10 runs, with the error bars denoting the standard error on the mean. The horizontal dashed line shows the result for the full BHC method.

Figure 1 shows how the adjusted Rand index (averaged over runs) varies with the randomised algorithm parameter, 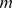. For 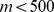, there is some loss in accuracy performance; however for 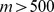, the adjusted Rand index is approximately that of the greedy algorithm.

**Figure 2:**
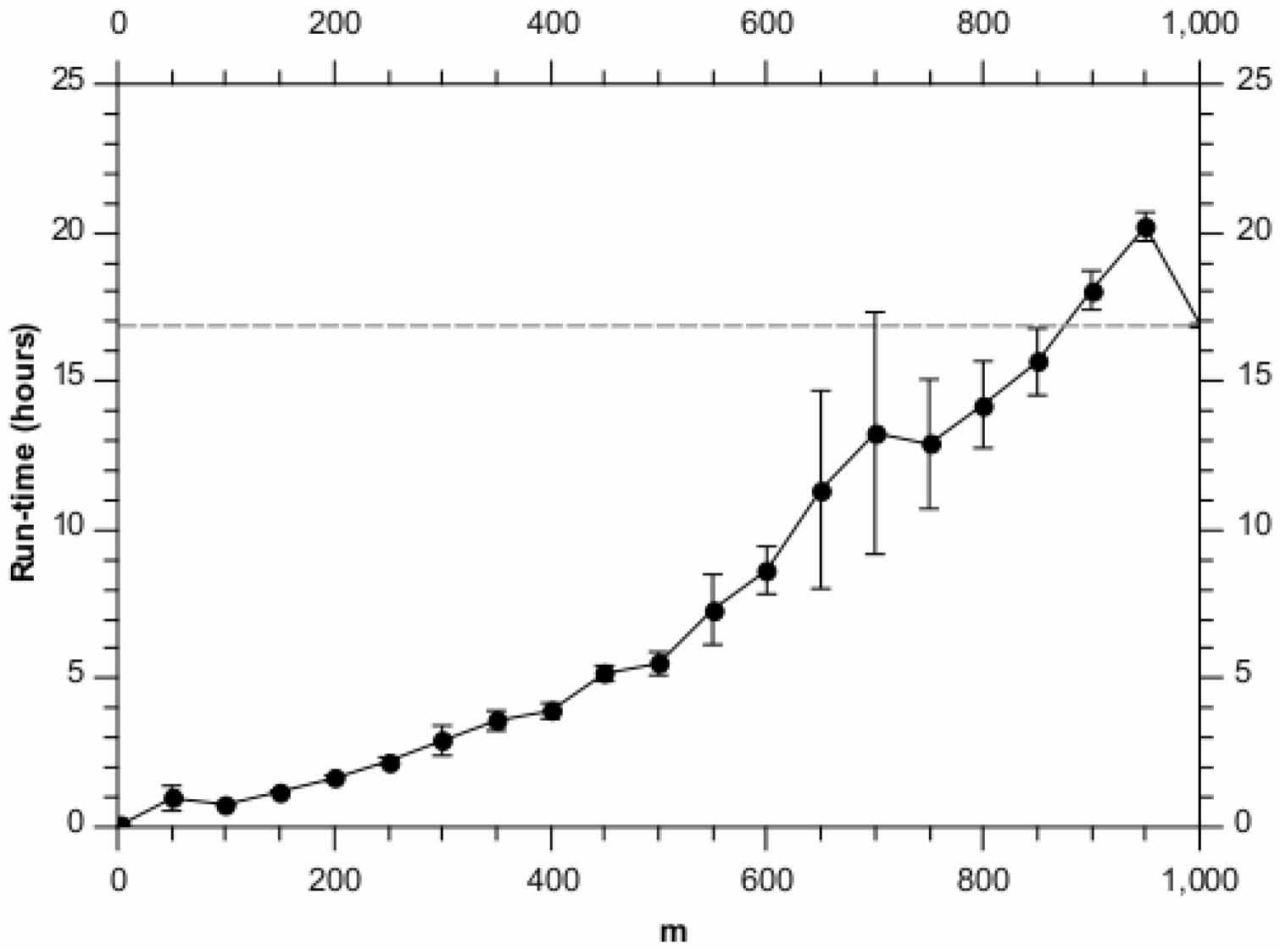
Run-times for different values of 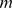, analysing the synthetic data set. Each point is the average of 10 runs, with the error bars denoting the standard error on the mean. The horizontal dashed line shows the result for the full BHC method.

Figure 2 shows the corresponding run-time performance. As expected, the algorithm is approximately linear in 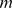 and a significant speed-up can be obtained over the greedy algorithm. For these synthetic data, one could therefore pick 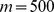 and get approximately the same performance as for the greedy algorithm, but with more than a 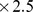 speed-up. And if some performance drop-off was acceptable, as much as an order of magnitude improvement is possible. We note that such a run takes only approximately 5 hours to complete on a single node 2.40 GHz Intel Xeon CPU.

**Figure 3:**
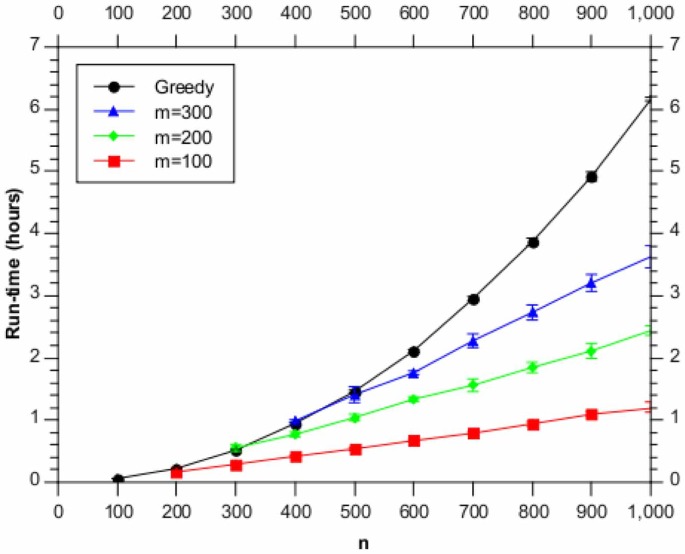
Run-time as a function of the number of genes, 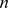, using (subsets of) the synthetic data. Shown are the results for 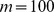 (red), 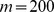 (green) and 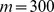 (blue), as well as for the full BHC method (black).

**Figure 4:**
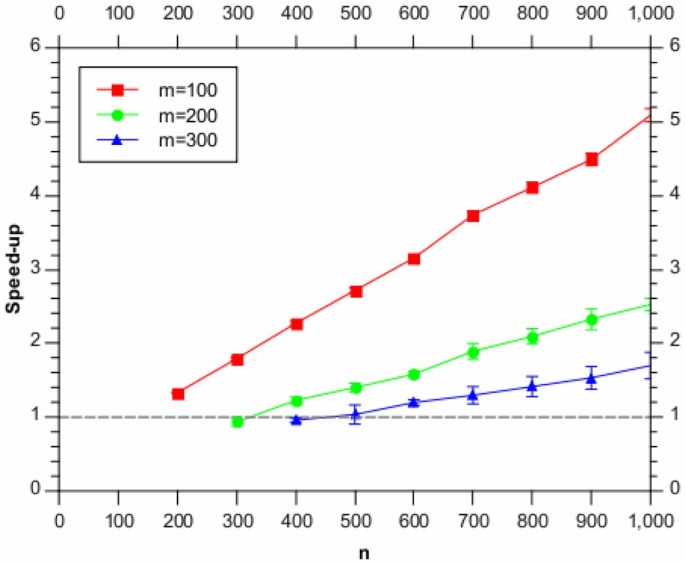
Speed up factor as a function of the number of genes, 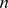, relative to the full BHC method, using (subsets of) the synthetic data. Shown are the results for 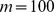 (red), 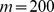 (green) and 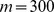 (blue). The horizontal dashed line shows the full BHC result.

We also consider how the run-time varies as a function of the total number of genes analysed, 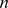. Figure 3 shows this variation for several different 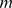 values. Figure 4 shows the same information, expressed a a speed-up over the greedy algorithm.

We note an interesting effect for the lowest value of 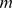 (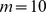) in Figure 1. A significant part of the performance degradation for lower 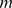 values in Figure 1 comes from the randomised algorithm over-estimating the number of clusters (these being synthetic data, we know the ground truth number of clusters). Investigation of the 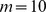 point shows that this effect is lessened for the synthetic data for small 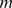. We believe that this is because for small numbers of data items, the inferred noise level is more weakly constrained. This in turn allows for clusters with higher noise levels, meaning the algorithm can explain the data using a smaller number of noisy clusters.

### Microarray Results

It is also important to validate the randomised algorithm on real microarray data. To do this, we use a subset of the data of [18], selecting genes that have a KEGG pathway annotation, using the version of the KEGG database to match that used in [20]. This consists of yeast cell cycle microarray time series for 1165 genes, measured at 17 time points.

As a performance metric, we choose the Biological Homogeneity Index (BHI) [21], as implemented in the R package clValid [22]. The BHI metric scores a clustering partition between 0 and 1, with higher scores assigned to more biologically homogeneous partitions with respect to a reference annotation set. This has proven to be an effective metric for measuring the performance of microarray-based gene clustering [14], [15].

**Figure 5:**
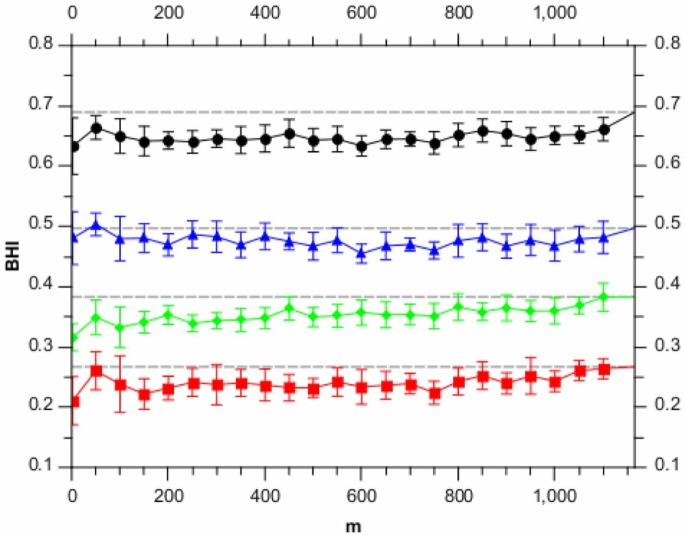
BHI scores for difference values of 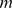, analysing the yeast microarray data set. Each point is the average of 10 runs, with the error bars denoting the standard error on the mean. The horizontal dashed line shows the results for the full BHC method. Shown are the results for the different gene ontologies, Biological Process (red), Molecular Function (green), Cellular Component (blue) and the logical-OR of all three (black). The BHI scores were all generated using the org.Sc.sgd.db annotation R package.

Figure 5 shows the BHI scores (averaged over 10 runs) as a function of the randomised-algorithm parameter, 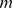. The BHI scores show very little variation for 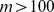, showing that the randomised algorithm is highly robust, in this case, to choice of 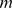. There is typically a small drop in performance relative to the greedy algorithm.

**Figure 6:**
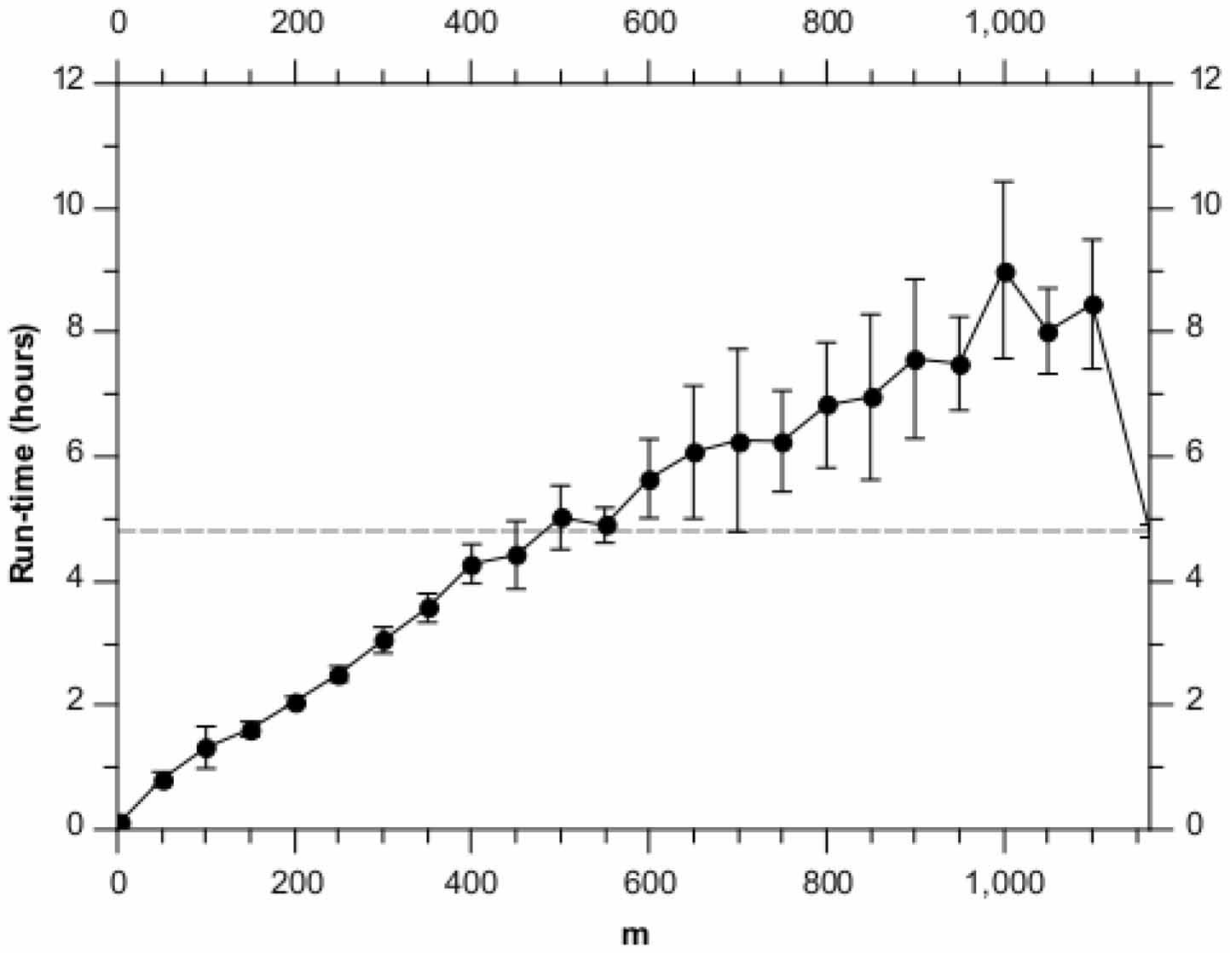
Run-times for different values of 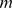, analysing the yeast microarray data set. Each point is the average of 10 runs, with the error bars denoting the standard error on the mean. The horizontal dashed line shows the results for the full BHC method.

Figure 6 shows the corresponding run-times. As with the synthetic data, we see the expected 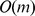 scaling. We note that here the overhead of the randomised algorithm means that for 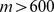 the greedy algorithm is actually faster. However, the BHI results in Figure 5 show that we could set 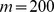 and gain almost a factor of 3 in speed while incurring only a minimal loss of performance. We note that such a run takes only approximately 2 hours to complete on a single node 2.40 GHz Intel Xeon CPU.

We note an interesting difference between Figures 2 and 6 in run time, relative to the greedy BHC algorithm. Because the number of genes is similar in both cases, one might expect the performance relative to the greedy algorithm to be similar. However (as is shown in these figures) the efficiency of the randomised BHC algorithm depends on how balanced (or otherwise) the dendrogram is. For example, if many levels of the dendrogram split into subsets of very different sizes (one big, one small), the randomised algorithm may have to go through many iterations in order to define the entire dendrogram. The run time is therefore dependent not only on the number of genes and time points, but also on the underlying clustering structure in the data. Essentially, unbalanced dendrograms make the randomised algorithm less efficient.

We also note that for 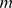 values close to the actual number of genes, it is a general feature that randomised BHC will tend to be slower than the greedy algorithm. This is because in this case, the randomised algorithm has to perform a greedy run with almost the entire set of genes to define the top branching of the dendrogram, and then assign all the genes to one of the two branches and run the greedy algorithm again for each of these subsets.

Figures 2 and 6 show an increased variance in the run time for 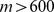. We believe this effect is due to the fact that for higher â€∼mâ€™ values, the run time is more likely to be dominated by a single run of the greedy algorithm for 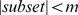 items. This will make the run time very sensitive to 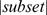, which will be affected by the randomisation of the overall algorithm.

## Discussion

We have presented a randomised algorithm for the BHC clustering method. The randomised algorithm is statistically well-motivated and leads to a number of concrete conclusions.

- The randomised BHC algorithm can be used to obtain a substantial speed-up over the greedy BHC algorithm.
- Substantial speed-up can be obtained at only small cost to the statistical performance of the method.
- The overall computational complexity of the randomised BHC algorithm is 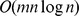.

The randomised BHC time series algorithm can therefore be used on data sets of well over 1000 genes.

Use of the randomised BHC algorithm requires the user to set a value of 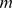. On the basis of the analyses presented in this paper, we recommend that a value of 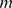 in the range 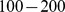 is reasonable, giving significant speed-up with minimal cost in terms of statistical performance.

The randomised time series BHC algorithm is available as part of the R package *BHC*, which is available for download from Bioconductor (version 2.10 and above) via http://bioconductor.org/packages/2.10/bioc/html/BHC.html.

We have also made available a set of R scripts which can be used to reproduce the analyses carried out in this paper. These are available from the following URL. https://sites.google.com/site/randomisedbhc/.

## Methods

In this section, we provide a mathematical overview of the time series BHC algorithm. Greater detail can be found in [15]. time series BHC combines the BHC clustering algorithm, coupled with a Gaussian process data model to provide a flexible, generative representation of microarray time series. Here we replace the standard (greedy) BHC algorithm with a randomised algorithm, improving the computational complexity of the method and hence its run time for scientifically-useful numbers of genes.

### BHC Algorithm

The BHC algorithm [13]–[15], [23] performs agglomerative hierarchical clustering in a Bayesian setting. In agglomerative clustering algorithms, each gene begins in its own cluster and at each stage the two most similar clusters are merged. BHC uses a model-based criterion to do this, also learning the most likely number of clusters given the data (something which many clustering methods are unable to do in a principled way). We note that the BHC algorithm can be interpreted as a fast approximate inference method for a Dirichlet Process Model (DPM) [13].

The prior probability, 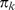, that a given pair of clusters, 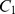 and 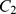, should be merged is defined by the DPM and is determined solely by the concentration hyperparameter for the DPM and the number of genes currently in each partition of the clustering. Bayes' rule is then used to find the posterior probability, 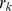, that the pair of clusters should be merged,
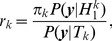
(1)where 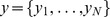 is the set of 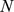 data points contained in clusters 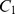 and 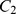. 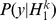 is the marginal likelihood of the data given the hypothesis, 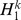, that the data 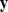 belong to a single cluster and requires the specification of a likelihood function, 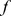, as the probabilistic model generating the observed data, 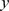. 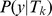 is the probability that the data could be partitioned in any way which is consistent with the order of assembly of the current partition and is defined recursively,
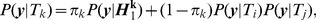
(2)where 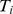 and 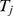 are previously merged clusters containing subsets of the data in 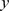.

When 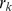 is greater than 0.5, it is more likely that the data points contained in the clusters 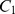 and 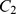 were generated from the same underlying function, 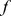, than that the data points should belong to two or more clusters. When 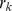 is less than 0.5 for all remaining pairs of clusters, the number of clusters and partitions best described by the data has been found.

For the purposes of the BHC algorithm, a complete dendrogram is constructed, with at each step the most likely merger being made. This allows us to see the log-probability of mergers in the whole dendrogram, even when this value is very small. To determine the likely number of clusters, given the data, we then cut the dendrogram wherever the probability of merger falls below 0.5 (i.e. non-merger is more likely).

As described in [13], the 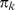 are dependent on a hyperparameter for the mixture model, 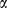. As in previous work on BHC, we set 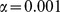 as a fixed value. This has the effect of setting a prior assumption of only weak clustering. One could learn this parameter as part of the BHC algorithm; we choose to not do this as it will substantially increase the run time of the algorithm.

The BHC algorithm provides a lower bound of the DP marginal likelihood, as shown in [13]. For the randomised algorithm, we note that the lower bound on the DP marginal likelihood is effectively determined using a subset of only 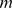 data items. These lower bounds are used in the usual way to optimise hyperparameters for each potential merger. One could attempt in principle to compute the lower bound using all 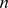 data items. However, this will be computationally intensive and so we do not consider it in this paper.

### Gaussian Process Regression

Gaussian processes define priors over the space of functions, making them highly suited for use as non-linear regression models. This is highly valuable for microarray time series [24]–[27], where a wide range of functional forms can be expected. In essence, Gaussian Process Regression (GPR) allows us to minimise the assumptions we must make as to the underlying structure in our time series data.

For the time series BHC model, we model an observation at time 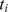 as 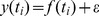. For each cluster, we assume the latent function 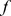 is drawn from a Gaussian process with covariance function 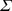, defined by hyperparameters, 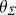. We also assume *iid* Gaussian noise, 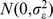.

Let 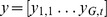 be the 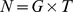 observations in a cluster of 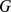 genes, where the 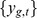 are time series of 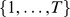 time points. Each gene is normalised to have mean 0 and standard deviation 1 across time points. The prior of 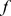 is given for fixed values of 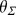, such that 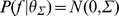. It follows that the likelihood function for 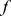 is 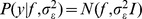, where 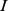 is the 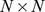 identity matrix. The marginal likelihood of the data, 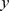, is then:
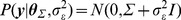
(3)

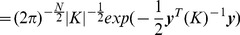
(4)where 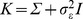 is the covariance function for 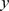.

Time series BHC implements either the squared exponential or cubic spline covariance functions. In this paper, we restrict our attention to the default choice of squared exponential covariance:
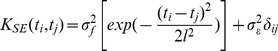
(5)where 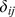 is the Kronecker delta function and 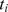 and 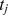 are two time points for 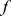. 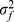 is the signal variance parameter for the covariance function and 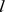 is the length-scale parameter.

### Randomised BHC Algorithm

To speed up the time series BHC, we implement the randomised BHC algorithm of [17] (specifically, algorithm 1). The key insight from which we hope to benefit is that the standard, greedy BHC algorithm is dominated by the computation of merges at the lowest level of the tree. Therefore, if we can reduce this load in a sensible way, it may be possible to produce a substantially faster algorithm.

Throughout this paper we will refer to the *top* of the dendrogram. This is the highest level of the dendrogram, where the whole set of genes is split into two subsets.

For reasonably balanced trees, the top levels should be well-defined even using only a random subset of the genes. From this idea, we can define the following randomised algorithm.

- Select a subset of 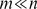 genes.
- Run BHC on the subset of 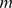 genes.
- Filter the remaining 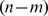 genes through the top level of the tree, computing merge probabilities between each individual gene and the two subsets of 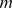 to decide to which branch the gene belongs.
- Including the original 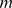 genes, we have now subdivided all genes on the basis of the top level branch of the tree.
- Now recurse for the gene subsets in each branch, until each subset size is 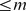, at which point use the standard BHC algorithm to complete the lower levels of the tree.

**Figure 7:**
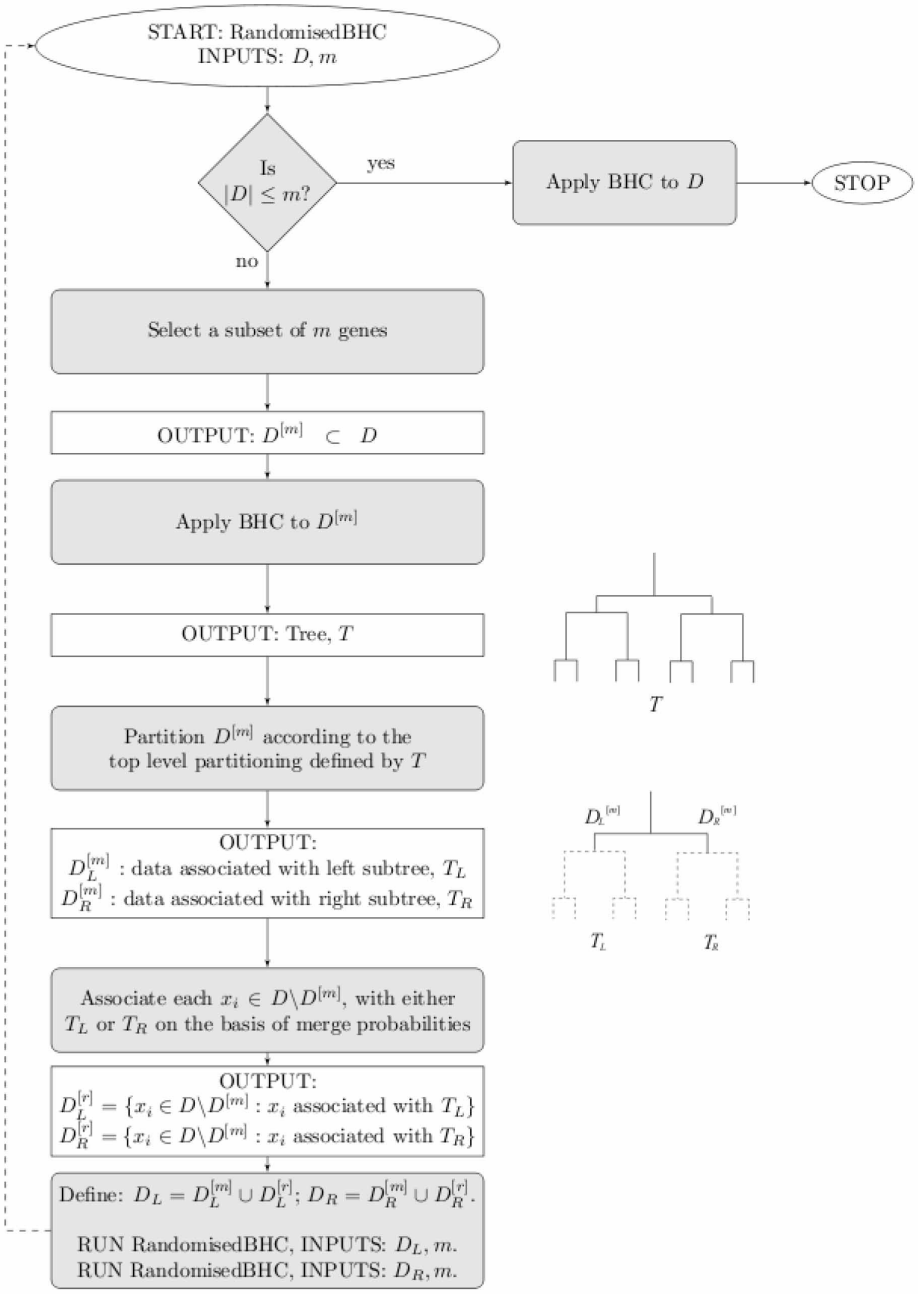
Flow chart showing the randomised BHC algorithm. The main loop is the randomised part of the algorithm, which is used recursively until the remaining gene subsets are small enough that it uses the greedy version of BHC to complete the tree and then terminates.

In effect, we are using estimates of the higher levels of the tree to subdivide the genes so that it is not necessary to compute many of the potential low-level merge probabilities. Figure 7 shows a flow chart describing the algorithm.

### Setting the Hyperparameters

The covariance function of the Gaussian processes used in this paper are characterised by a small number of hyperparameters. These hyperparameters are learned for each potential merger using the BFGS quasi-Newton method [28].

This merge-by-merge optimisation allows each cluster to have different hyperparameter values, allowing for example for clusters with different intrinsic noise levels and time series with different characteristic length scales.

### Utilising the Covariance Matrix Block Structure

We assume in this paper that each time series is sampled at the same set of time points. This leads to a block structure in the covariance matrix, which can be utilised to greatly accelerate the computation of the Gaussian process marginal likelihood.

The computational complexity of BHC is dominated by inversion of the covariance matrix. Considering the case of a group of 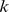 genes, each sampled at the same 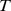 time points, the naive approach to matrix inversion would require us to invert a 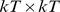 matrix, which is an 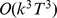 operation. However, we can instead use block matrix pseudoinversion, which recursively reduces the block size to one, at which point the remaining inversion is an 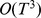 operation.

We also note that this is equivalent to a Bayesian analysis using a standard multivariate Gaussian. Indeed, considering the task in this way may be a simpler way of doing so and is certainly a useful way of gaining additional insights into the workings of the model.

### Computational Complexity

When proposed merges have constant cost (the case considered by [17]), the standard greedy BHC algorithm has 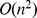 computational complexity.

For the time series BHC algorithm however, the merges do not have have constant cost. For a given node, we are merging 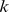 gene time series, each of length 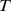. We therefore have to consider a 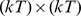 covariance matrix, which we must invert. As noted in [15], this matrix is actually a block matrix consisting of 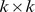 blocks, which means we can invert it in 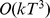 operations.

Because 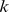 will be as large as 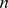 for the merges closer to the root node of the tree, this gives the greedy time series BHC algorithm a worst-case computational complexity of 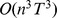.

The randomised algorithm for case of constant cost merges has 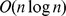 complexity [17]). Heller and Ghahramani show that, for reasonably balanced trees, the complexity is dominated by the filtering step. Each of the 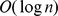 filtering steps is 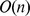, resulting in the overall 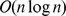 complexity. For the time series BHC algorithm, the filtering step is 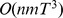, because of the additional cost of merging time series clusters. As in the original analysis there will be 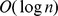 filtering steps, giving an overall computational complexity for the randomised version of time series BHC of 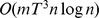.
